# Memory for medicinal plants remains in ancient and modern environments suggesting an evolved adaptedness

**DOI:** 10.1371/journal.pone.0258986

**Published:** 2021-10-25

**Authors:** Joelson Moreno Brito Moura, Risoneide Henriques da Silva, Washington Soares Ferreira Júnior, Taline Cristina da Silva, Ulysses Paulino Albuquerque

**Affiliations:** 1 Departamento de Biologia, Universidade Federal Rural de Pernambuco, Recife, Pernambuco, Brazil; 2 Departamento de Botânica, Universidade Federal de Pernambuco, Recife, Pernambuco, Brazil; 3 Laboratório de Investigações Bioculturais no Semiárido, Universidade de Pernambuco, Petrolina, Pernambuco, Brazil; 4 Departamento de Biologia, Universidade Estadual de Alagoas, Santana do Ipanema, Alagoas, Brazil; University of Padova, ITALY

## Abstract

Adaptive memory is the propensity of human memory to easily store and retrieve important information to deal with challenges related to the Pleistocene. Recent evidence shows that humans have had a multiregional evolution across the African continent, including the rainforests and deciduous forests; however, there is little evidence regarding the implications of these origins and the relevant and recurring challenges of these environments on survival processing advantage in memory. In this study, we conducted an experiment with volunteers to analyze whether adaptive memory operates in the retrieval of important information to solve challenges of using medicinal plants to treat diseases in the ancestral environments of the savanna, rainforests, and deciduous forests compared to the modern environments of desert, tundra, coniferous forest, and urban areas. We used simulated survival environments and asked volunteers (30 per simulated scenario) to imagine themselves sick in one of these environments, and needing to find medicinal plants to treat their disease. The volunteers rated the relevance of 32 words to solve this challenge, followed by a surprise memory test. Our results showed no ancestral priority in recalling relevant information, as both ancestral and modern environments showed a similar recall of relevant information. This suggests that the evolved cognitive apparatus allows human beings to survive and can create survival strategies to face challenges imposed in various environments. We believe that this is only possible if the human mind operates through a flexible cognitive mechanism. This flexibility can reflect, for example, the different environments that the first hominids inhabited and the different dangerous situations that they faced.

## Introduction

Adaptive memory refers to cognitive adaptation based on survival that prioritizes the storage and retrieval of important information in human memory to deal with recurring challenges related to the Pleistocene, 2,5 millions of years ago—that is, ancestral priority in the functioning of memory [1]. This may reflect the importance of ancestral environments in the evolution of most human psychological mechanisms (see [2]), enabling better mnemonic performance to occur when induced by problems present in ancestral settings, such as escaping predators and treating infections (see [3, 4]). This adaptive memory bias leads to greater information retention, regardless of the specific proximate mechanisms that may be involved [1]. In this sense, a mnemonic adaptation refers to the selective pressures that shaped a memory that helped early human beings to solve adaptive problems and, therefore, increased the chances of survival and reproduction [5].

For example, it was observed that situations involving the search for medicinal plants for treating an infection in a savanna ancestral environment promoted survival advantage in memory processing compared to the modern environment [3]. Other studies analyzing this mnemonic adaptation also obtained similar results (see [5–10]), including studies among people living in different environmental contexts [11, 12]. In this sense, the book published by Schwartz et al. [13] gathers several theoretical and empirical works about the adaptive nature of memory. In addition, remembering the location of food, the habitat of a predator or a partner available to mate facilitated the survival and reproductive success of hominids [3, 14, 15].

These mental adaptations were shaped by the way hunter-gatherer hominids solved recurrent adaptive problems in ancestral environments [2]. However, recent evidence shows that human beings have undergone a multiregional evolution across the African continent and in other regions of the planet [16–20], with little evidence on the implications of these origins on adaptive memory (see [15, 21]). For instance, a study by Yang et al. [15], showed that important information for survival is remembered by people both in ancestral survival scenarios (savanna-like pastures) and in non-ancestral/modern environments (mountains). Moreover, a study by Silva et al. [22] provided evidence that the previous experience of people with common illnesses and the frequency of these illnesses intensifies the memorization and retrieval of important information to deal with these illnesses. This suggests that the human mind can adaptively respond well to recurrent threats in the current environment. Therefore, it is reasonable to consider that adaptive memory can operate in recent environments and in other evolutionary environments besides the savanna, such as in humid and dense tropical environments; however, this has not been tested so far.

During evolutionary history, the first human beings had to constantly interact with the environment, using natural resources to obtain nutrients or avoiding dangers and threats from predators. This indicates that their interactions may have affected our cognitive apparatus such that the human mind has adapted and developed psychological mechanisms responding to a wide range of challenging situations [2]. Recurrent selection pressures in the *environment of evolutionary adaptedness*, acting over evolutionary time on a regular basis, boosted the construction of mental adaptations in the first humans to solve lasting adaptive problems [2]. Environment of evolutionary adaptedness does not refer to a specific environment or time; it is a set of statistical factors of recurrent selection pressures or cause and effect relationships that drove the increase in the frequency of certain alleles underlying an adaptation, until they became typical species or promoted a frequency-dependent balance [2]. According to Tooby and Cosmides [2], evolutionary processes are slow and require thousands of years to develop complex cognitive adaptations; therefore, the human mind remains adapted to the Pleistocene savanna. However, this concept does not consider two important aspects of the evolutionary history of *Homo sapiens*: i) human evolution and development may have occurred in various environments during the Pleistocene [18, 20]; and ii) human activities can accelerate biological evolution, thus modifying genetically inherited predispositions (see [23]). These aspects may even reflect flexible mental adaptations, which integrate the mechanisms molded in paleoenvironments with mechanisms built during the individual’s ontogenetic development [24]. For example, an idea proposed by Orians [25] assume that exist the predisposition of human beings to prefer savanna environments, due to the importance of this environment during evolutionary history, which, being an environment with sparse trees that allowed a panoramic view, facilitated, among other things, the observation of the approach of predators, which helped in the survival and reproduction of hominids. However, this preference is not observed in some cultures, which may result from the establishment of human beings in different modern environments [26, 27]. In addition, the study by Soderstrom and McCabe [4] brought evidence that the good performance of recall occurs in modern (city) and ancestral (pasture) scenarios, suggesting that in some situations there is no ancestral priority in the survival processing advantage in memory.

In this sense, based on recent paleoanthropological evidence from the fossils of the first human beings [18, 20, 28, 29], we consider the rainforest, deciduous forests, and the savanna asancestral environments. For example, recent fossil evidence suggests that *H*. *sapiens* may have originated and evolved in distinct regions throughout the African continent (see [17, 19, 30]). The first hominids adapted to various environments during the Pleistocene over a wide latitudinal range, such as the temperate north (which includes deciduous forests) the subtropical region of China, and in the tropical regions of Southeast Asia [18, 20, 29]. At the end of the Pleistocene, some *H*. *sapiens* had already occupied the cold temperate environments of the Arctic region [31]. Richerson and Boyd [31] even argue that the long and slow evolutionary history of human beings in different environments may have driven the growth and development of the brain as we know it today, because the human construction of subsistence systems adapted to spatial variation.

In this study, we analyzed the performance of human memory in situations of survival in the ancestral savanna, rainforest, and deciduous forest environments, and in the modern coniferous forest, desert, tundra, and urban environments, to investigate whether an ancestral priority of adaptive memory really exists. The modern environments considered here are regions in which there has been no paleoanthropological evidence about hominid origin and evolution until now. This study can help to elucidate the controversial results in literature that may reflect the conception of evolutionary psychology suggesting that we evolved in a specific environment (savanna) and that is why we adaptively respond better to challenges related to this biome, not considering, for example, the ancestral environment of the rainforest.

For this, we carried out an experiment adapted from Nairne et al. [1] in which we simulated both ancestral and modern survival scenarios. In the typical survival processing paradigm, participants imagined that they were trapped in a savanna without any survival materials and must face potential dangers, and that they needed to evaluate a list of words and their relevance to survive in the relevant scenario [32]. In this study, we asked volunteers to assess the relevance of information to solve the recurring challenges imposed in these scenarios and later subjected them to a surprise memory test. The memory test allowed us to analyze whether the relevant information was remembered when related to ancestral environments.

If human evolution was in fact multiregional, it is reasonable to consider that adaptive memory is a reflection of an evolutionary history that has trodden several paths, with hominids venturing and adapting to threats that exist in hot, dry, and sparse environments as well as in humid and dense environments. Therefore, these evolutionary paths must have some effect on adaptive memory. We suggest that the understanding of how adaptive memory operates can be more complete with the expansion of its analysis in a manner that encompasses rainforests as well as modern environments.

Thus, we tested the hypothesis that there is an ancestral priority in the cognitive strategies mobilized in the human mind to find medicinal plants to treat diseases. The challenge related to the search for medicinal plants for treating diseases is an interesting model for a broader understanding of adaptive memory, as this was a basic recurrent challenge essential for the survival and evolution of hominids in ancestral environments [3, 20, 33–35]. In our experiment, we predicted that, on average, people would significantly remember the words classified as most relevant to finding and using medicinal plants in the simulated ancestral survival scenarios (savanna, rainforest, and deciduous forest) when compared to the modern scenarios.

There is evidence that *Homo sapiens* and *H*. *neanderthalensis* have used several parts of different plants for medicinal and technological purposes [36, 37]. Situations of parasite infections or invasion by predators during foraging activities may have stimulated the first humans to look for medicinal plants [38]. In addition, *Homo habilis* used medicinal plants to treat diseases based on observation of plant-based self-medication by other animals [39]. The use of medicinal plants by non-human primates may also involve different plant parts. For instance, some orangutans (*Pongo pygmaeus*) in Indonesia ingest *Dracaena cantleyi* leaves for the treatment of parasites [40]. Although the therapeutic use of plants is understood to be essential for the survival and evolution of hominids, the events that led to this behavior are still uncertain, with only few hypotheses about its origin (for a complete argument see Albuquerque et al. [33]).

In this sense, we conducted an empirical experiment that analyzed how human memory stores and retrieves the relevant information to challenges related to the use of medicinal plants in different environments. We used the same challenge related to medicinal plants in all simulated environments because using qualitatively different survival threats (for example, escape from a predator in the savannah *versus* escape from a murderer in the city) could lead to different levels of threat meaning [41], that is, one threat can be perceived as more dangerous than the other. Therefore, our aim was to obtain evidence about how human memory may have evolved to be versatile in solving the problems imposed by ancestral and recent environments.

## Materials and methods

### Experiment and word selection

We designed an experiment, adapted from the work of Nairne et al. [1], analyzing the survival processing paradigm, in which we simulated different survival scenarios wherein each volunteer evaluated the relevance of information to perform the task of finding and using medicinal plants to treat a disease. We do not use a control scenario in our experiment. Although, we chose to only use scenarios related to a survival situation encompassed ancestral environments, considering that the task of looking for medicinal plants to treat diseases activates the advantage of survival processing in memory (see [3]). The scenarios encompassed ancestral environments—rainforests, deciduous forests, and savanna—and modern environments—coniferous forests, deserts, tundra, and urban. We maintained the same survival conditions in all environments. The objective was to analyze whether the most relevant information would be quantitatively more recalled in a free recall surprise test. We predicted that relevant information would be best remembered when the challenge was present in ancestral environments.

The "information" in our experiment is represented by words (concrete nouns) that act as stimuli in the tests of memorization (see [1]). The relevance of this information was decided by the volunteers themselves, as even seemingly irrelevant stimuli for fitness, such as a pencil, could become relevant depending on the situation and a jacket could be relevant depending on the location [42]. Relevance was measured using a Likert scale that ranged from 5 for extremely relevant to 1 for extremely irrelevant. In the recall test, our objective was not to analyze the word itself, but rather if the relevance given to each word would influence its recall depending on the environment, regardless of the meaning of the information.

Thus, we randomly selected 32 words that were unrelated concrete nouns, based on the free association norms for Brazilian Portuguese words in memory studies, which include 1004 standardized words [43]. The selection criterion was based on two aspects: i) concrete nouns, as they generate less confusion in free recall tests; and ii) unrelated nouns that have little or no semantic relationship, as this relationship can be a confusing factor in the analysis. The 32 selected words that we used as a stimulus, followed by their translation into English, are described in S1 Appendix. The same set of 32 words was used for all simulated survival scenarios. The words were randomized before being presented to each participant.

### Survival scenarios

To test whether there is an ancestral priority in the cognitive strategies mobilized in the human mind, we use simulated survival scenarios that are dangerous situations (see [1, 33]), but made some adaptations. In adaptive memory studies, survival scenarios usually cover only the ancestral savanna environment and the modern city and mountains, among others. However, to test our hypothesis, we used the other five great terrestrial biomes, considering the rainforest and deciduous forest as ancestral environments, according to the classification by Odum [44], to represent the scenarios of survival, maintaining the same situation of danger in all the scenarios. In addition, we displayed an image of the landscape corresponding to the respective environment to each participant so that the situation had another associated stimulus. The use of images is common in psychology studies that analyze, for example, the preference for landscapes by human beings, as carefully selected images promote a good representation of the actual environment (see [27]). In this study, we used seven images used by Moura et al. [27]. We describe all seven simulated scenarios used in S2 Appendix. Below is an example of one of the simulated scenarios used in the experimente:

Scenario: “*Imagine that you are alone and sick in a rainforest*, *without basic materials for survival*. *In the coming days*, *you will need to find and use medicinal plants to treat this disease*. *We will show you a list of words*, *and would like you to assess the relevance of each of these words in your attempt to treat the disease and to survive in this environment*”.

### Participants

We recruited 210 volunteers (age mean = 22.4 years; *SD* = 4.07), all students from the Universidade Federal Rural de Pernambuco, Brazil, including 51% women and 49% men, with the objective of analyzing how adaptive memory operates in different simulated survival environments with challenges related to the use of medicinal plants. Volunteers were recruited through direct contact with each volunteer. Before the experiment, the following question was asked: "*Do you know what medicinal plants are*?", with answer options as "yes" or "no". Volunteers who did not know what medicinal plants were were excluded from the study, as not knowing the model used as a challenge (medicinal plants) could bias the experiment. New recruitments were made to replace these participants.

The Research Ethics Committee involving human beings at the Universidade de Pernambuco approved this study (decision number 2.944.271). All participants read and signed the Free and Informed Consent Form, which explained the procedures and objectives of the research.

### Procedure

**Fig 1:**
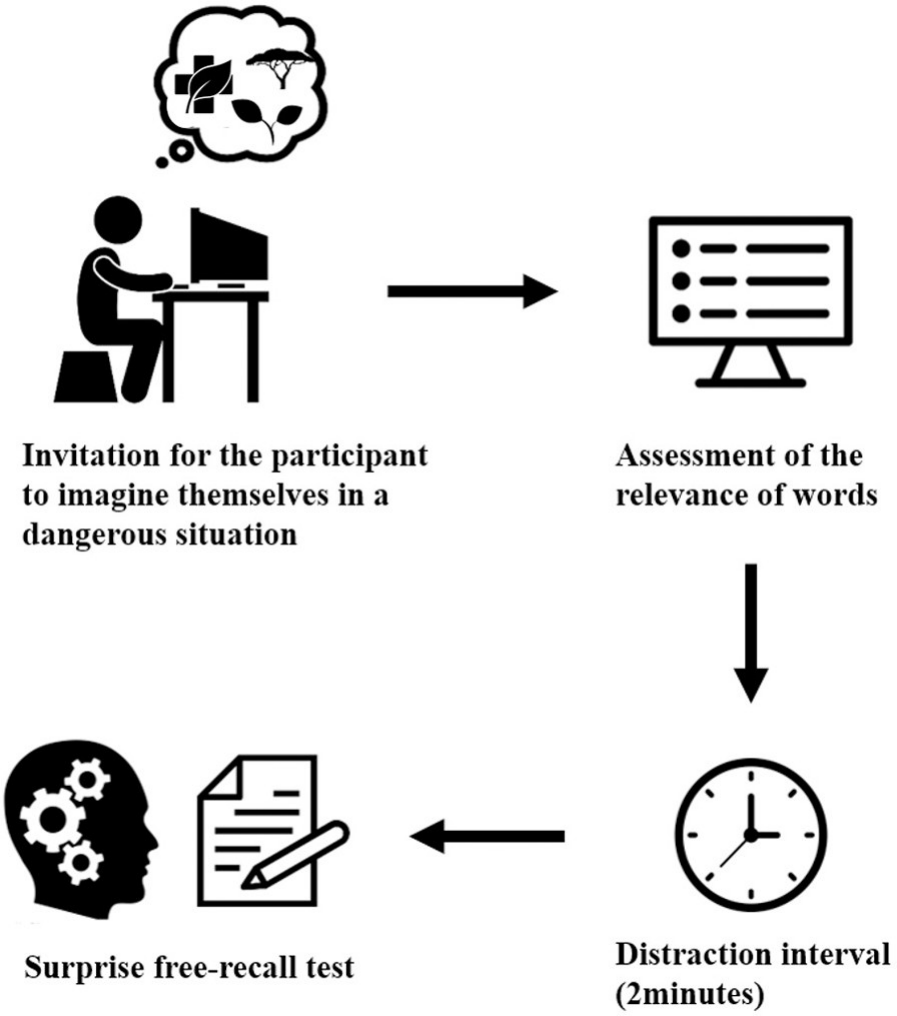
Scheme of the experiment used to analyze the functioning of adaptive memory in simulated situations of danger in ancient and modern environments.

Participants were tested individually in an isolated room without any external interruption. The volunteers were divided into seven groups (n = 30 in each group) that differed by the type of simulated environment. These environments were considered ancestral—rainforest, deciduous forest, and savanna—and modern—coniferous forest, desert, tundra, and urban. The objective was to observe how information processing in memory varies depending on the type of environment. In the first part of the experiment (Fig 1), a simulated survival scenario was presented to the participants, and each participant was instructed to assess the relevance of 32 words to survive in the presented danger situation. Each word was exposed on a computer screen for 5 seconds. Immediately after that, there was a 2-minute distraction interval, which is the time necessary to avoid the tendency to remember the first elements of a list (primacy effect) and the last elements (recency effect), during which the participants filled out a form about demographics. After that, a surprise test of free recall was made, in which the participants were instructed to write the maximum number of evaluated words that they could remember on a sheet of paper, regardless of the order, within 10 minutes. Each experiment had a total duration of 30 minutes. We expected that the most relevant words were retrieved in the memory test when related to the ancestral environment.

### Data analysis

To test our hypothesis that there is an ancestral priority in the cognitive strategies mobilized in the human mind to find medicinal plants to treat diseases, we developed mixed generalized linear models (GLMs) with binomial distribution, using the *R lme4* package [45]. As the environment and relevance of the information are factors that influence memory in the recall test, the dependent variable was the *recall* of the words evaluated by the participants. Therefore, the independent variables were the types of simulated *environments*, the words’ *relevance*, and the *interaction* between these variables. As each participant has a different memorization capacity, we included the participants as a random factor to discard the effect of the difference in memorization among the three models developed. The effect size of the variables was measured using the R broom.mixed package [46]. All analyses were performed in the R version 3.6.1 environment [47].

We adjusted the model to obtain the lowest AIC value, as proposed by Agresti [48]. Although the variable relevance when isolated, was not significant (assuming a significance level of p < 0.001), we included it in the adjusted model (AIC = 9013.2), as analysis of the more complex model showed significant interactions between the variables (relevance + environment) that influenced the recall. For Agresti [48], when the variables interact significantly, the variables that make up this interaction should not be removed from the model. The resulting model explains about 4.5% of the variation observed in the recall (R2 marginal = 0.044). We also created a graph to observe the relationship between the variables using the ggplot2 package [49].

## Results

The relevance of the words influenced their recall by the participants (n = 210, which was 30 per scenario) in the modern urban, tundra, and desert environments, in addition to the ancestral environments of the savanna and rainforests (*p* < 0.001). The analysis of our most complex model, which had a better fit (AIC = 9013.2), showed that the interactions between the relevance and environment variables had a significant effect on the model.

**Fig 2:**
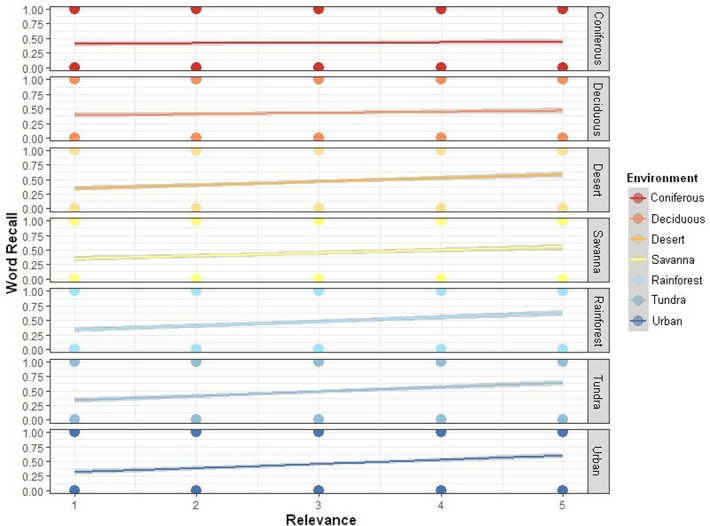
Relationship between the relevance + environment interaction and the recall of the participants. The colored lines represent the regression line of the relationship between the variables. The regression line inclines significantly in the desert, savanna, rainforest, tundra, and urban environments. The more relevant a word is, the more the person retrieves this information in the desert, rainforest, tundra, savanna, and urban environments.

We observed that the following interactions were significant: relevance + desert (*z* = 3.893; *p* = 9.90e-05), relevance + savanna (*z* = 3.008; *p* = 0.00263); relevance + rainforest (*z* = 4.694; *p* = 2.67e-06); relevance + tundra (*z* = 5.042; *p* = 4.61e-07); relevance + urban (*z* = 4.447; *p* = 8.72e-06) (Table 1) (Fig 2). This means that the greater the relevance of information, which ranged from 1 to 5 on the Likert scale—to use medicinal plants to treat a disease in a given environment—in this case, the desert, savanna, rainforest, tundra, and urban environments—the more it is recovered in the memory test. However, the relevance + deciduous (*z* = 0.932; *p* = 0.35126) and relevance + coniferous (*z* = 0.784; *p* = 0.43313) interactions were not significant. The interactions show that for each unit of increased relevance, the chances of a word being remembered in the tundra environment are increased by approximately 35% (OR = 1.35), in the rainforest by 32% (OR = 1.32), in the urban environment by 30% (OR = 1.30), in the desert by 25% (OR = 1.25), and in the savanna by 19% (OR = 1.19) (Table 1). Each of the 32 words used in the experiment is presented in S1 Appendix, along with descriptive information on the average classification of each word and the recall ratio (95% confidence interval) when processed in each environment.

**Table 1:** Results of binomial GLM, estimated regression parameters, standard errors, z values, p values and effect size (in Odds ratio) estimated for relevance in each of the interactions (environments + relevance) that we used in the model, accompanied with the respective confidence intervals.

Interactions (relevance + environment)	Estimate	Std. Error	z value	Pr(>|z|)	Odds ratio	Low conf. (2.5%)	High conf. (97.5%)
(Intercept)	-0.41649	0.13872	-3.002	0.00268	0.659	0.502	0.865
Coniferous forest	0.03188	0.04068	0.784	0.43313	1.03	0.502	0.865
Deciduous forest	0.05292	0.05677	0.932	0.35126	1.05	0.943	1.18
Desert	0.22640	0.05816	3.893	9.90e-05	1.25	1.12	1.41
Savanna	0.17676	0.05876	3.008	0.00263	1.19	1.06	1.34
Rainforest	0.27559	0.05870	4.694	2.67e-06	1.32	1.17	1.48
Tundra	0.29955	0.05941	5.042	4.61e-07	1.35	1.20	1.52
Urban	0.26235	0.05900	4.447	8.72e-06	1.30	1.16	1.46

### Comparison of word evaluations and the proportion of recall between environments

We performed an additional Kruskal–Wallis test (non-parametric test) to compare the word evaluation and the proportion of recall between all survival scenarios. This test was chosen because our data had a non-normal and heteroscedastic distribution.

The comparison of word evaluation values showed that there were no significant differences in the relevance given to the words in each environment (*H* = 1.12; *p =* 0.98). The descriptive analysis results of the words assessment are shown in Table 2.

**Table 2:** Median differences (Kruskal–Wallis) and the descriptive analysis of the words rating in the survivor scenarios.

Survival scenarios	Median	Mean	Standard deviation
Coniferous forest	2	2.76	1.63
Deciduous forest	3	2.86	1.68
Desert	3	2.79	1.63
Savanna	3	2.78	1.58
Rainforest	3	2.76	1.62
Tundra	3	2.82	1.60
Urban	3	2.99	1.59

Regarding comparison of the proportion of words remembered in each environment, there were also no significant differences (*H* = 1.42; *p =* 0.96). The descriptive analysis results of the proportion correct recall are shown in Table 3. Thus, both the evaluation and the proportion of word recall were similar between the environments. However, as noted in the GLMM analysis, the greater the relevance of information in the tundra, rainforest, urban, desert, and savanna environments, the more are its chances of recovery.

**Table 3:** Median differences (Kruskal–Wallis) and the descriptive analysis of the proportion correct recall in the survivor scenarios.

Survival scenarios	Recall	Median	Mean	Standard deviation
Coniferous forest	0.42	12	12.59	5.09
Deciduous forest	0.41	12.5	12.75	5.31
Desert	0.43	11.5	13.46	6.84
Savanna	0.45	13	13.06	6.32
Rainforest	0.47	14.5	13.87	7.09
Tundra	0.47	11	14.18	7.34
Urban	0.45	13.5	13.62	6.80

## Discussion

This study comprised an analysis of the functioning of adaptive memory in different simulated environments rejected the hypothesis that there is only ancestral priority in the way it operates, i.e., it is not restricted only to the savanna, rainforests, and deciduous forests. We provided evidence that relevant information to search for medicinal plants in the desert, rainforest, and tundra environments was also recovered in memory. Based on our evidence, human cognition may have been selected slowly over evolutionary history in response to recurring challenges, present in diverse environments explored by hominids, such as the use of medicinal plants to treat diseases. According to Nairne and Pandeirada [50], the effects of survival processing on memory may be acting through a general survival optimization system that deals with varied challenges, selected for having helped human beings to face recurrent threats both in ancestral as well as modern environments. For example, the effects of survival processing were observed in different simulated scenarios, such as “city” and even in “outer space” [21] and in situations involving zombie threats [51].

In this sense, the human mind adaptations exist because they helped to solve recurrent and relevant adaptive problems to survival or reproduction of the hominids during evolutionary history (see [2]). Since in our study in the deciduous forest and coniferous forest environments there was no significant interaction with the relevance of the information, this may reflect the absence or less occurrence of certain challenges in these environments. However, this needs to be analyzed in future studies.

In fact, the environments explored by hominids are not restricted to the savanna of the African continent and may include other biomes (see [18, 20, 31]). For example, there is evidence that the oldest hominid, *Graecopithecus freybergi*, inhabited a savanna environment in the region of Greece, between 7.37 and 7.11 million years (Ma) [16]. In addition, according to Zhu et al. [29], fossil evidence indicates that hominids dispersed from the African continent towards East Asia at least 2.1 Ma. This suggests that even at the beginning of the Pleistocene, hominids entered Asia, adapting to different geographic and climatic configurations [52].

For a long time, there was a consensus that *H*. *sapiens* originated in South Africa, but fossil evidence involving recent discoveries in the Gruta da Aroeira in Portugal [53], added to the evidence that *H*. *sapiens* inhabiting the Morocco region had a mixture of *H*. *sapiens* fossil characteristics from other parts of Africa, suggest a multicentric genesis for our species [17, 19]. Therefore, this evidence suggests that several regions may have been important during the evolution of humans.

Although the savanna is still considered the main environment of human evolution, fossil evidence indicates that the origin and large divisions of hominids may have occurred outside Africa (see [16, 30, 54]). There is even evidence of hominid adaptations in humid and dense environments. Among these adaptations, we can include the use of fire [28], and traces of foraging activities in the interior of the rainforest [55]. The idea of hominid origin and evolution in rainforests is not new. Andrews [56] had already proposed the possibility of the first hominids to have evolved in both closed and open forests. This proposition has gained strength as new paleoanthropological evidence of human evolution in the rainforests has emerged (see [18, 20]).

In this sense, given that cognitive mechanisms result from responses to relevant and recurring selective pressures from ancestral environments, a challenge for evolutionary psychologists would be to expand their analysis in a way that covers the adaptive problems of different environments in which we evolve and the influence of the evolved psychological mechanisms in solving problems different from those found only in the African savanna [57]. For example, dealing with infections was a challenge in several environments during the Pleistocene, and success in this, regardless of the environment in which such an adaptive problem was present, may have been crucial for the survival and reproduction of hominids [33]. This would help explain, for example, why Yang et al. [15] found that important information for survival is recovered both in ancient survival scenarios as well as in modern environments. The work by Silva et al. [22] also showed that the challenges encountered in current environments are important for our cognition.

We do not deny the important role of the ancestral past in the construction of the human mind, but it is essential to abandon the idea that we are still mentally predisposed to the ancestral environments of the Pleistocene. We provide evidence that human respond adaptively to challenges related to the use of medicinal plants present in ancient as well as modern scenarios. In this case, we assume that human beings have inherited adaptations from their hominid ancestors; however, human activities carried out in different environments over time can accelerate biological evolution and may modify genetically inherited predispositions (see [23, 27, 58]). Barrett [24] argues that certain adaptations can provide plasticity to the human mind. According to the author, it is possible that the human mind integrates both general and specific mechanisms, which were shaped during evolutionary history and by the individual’s ontogenetic development. As per Barrett [24], the cognitive processes of human beings can operate through mechanisms of heterogeneous origin, with new structures evolving from older structures and ancestral characteristics combined with relatively recent characteristics. This aspect of human cognition may have been important in reducing the incompatibility between ancestral and modern environments.

According to Tooby and Cosmides [2], evolutionary processes are slow and require several generations to develop complex psychological mechanisms. This argument supports the idea that we still act in the face of imposed environmental problems in a similar manner to our hominid ancestors during the Pleistocene (see [59]). A classic example is the preference for fatty and sweet foods, which is behavior adapted to ancestral environments with little fat availability, but poorly adapted in the current environment, thus increasing the incidence of cardiovascular illness [59]. However, there is recent robust evidence of significant genetic changes in human populations [58, 60, 61]. Although previous examples do not involve changes in cognitive aspects, it is probable that psychological mechanisms evolved in the Pleistocene may be subject to evolutionary processes that promote rapid changes in a few generations, being able to generate new cognitive structures that used “model” older mechanisms to adapt to today’s environments challenges (see [24]). In this sense, evidence that people remember important information in modern environments, may indicate that there is no incompatibility, and that genetically inherited psychological mechanisms can be modified and adjusted to the current environment as well.

Our study provides evidence that adaptive memory can operate through a flexible and adaptive cognitive mechanism, as argued by some other scientists as well [14, 15, 22, 24, 62–64]. However, the absence of a control scenario in our study limits the interpretation of our results, making it important to analyze the mnemonic performance in other survival scenarios compared to a control scenario. From an evolutionary point of view, memorizing information related to a wide range of environments and recurring danger situations over the generations may have given the first hominids greater ability to survive.

If this is true, cognitive adaptations do not just respond to situations related to a specific environment. The first hominids may have evolved psychological mechanisms, reusing pre-existing mechanisms, which adjusted to different environments during their evolution, not just being restricted to the Pleistocene era [24, 31]. Thus, future studies should investigate the factors leading to certain environments and not just ancestral environments, to intensify the effect of survival processing advantage on memory and to determine the cognitive mechanisms that act as stimuli for information prioritization in memory.

## Conclusion

The cognitive apparatus selected throughout evolution allows humans to survive and create survival strategies to face recurring challenges in various environments. Retrieving important information for survival related to the use of medicinal plants can be one of these strategies. We suggest that this is only possible if the human mind operates through an evolved versatility that gives it flexibility. This flexibility can reflect, for example, the different environments that the first hominids inhabited and the different and recurring situations of danger that they had to face.

### Limitations and future research

The main limitation of this study was that the performance of memory was not compared to a control condition (for example, “moving” scenario commonly used in studies that analyze the survival paradigm). Therefore, this makes it difficult to understand whether the mnemonic performance was really good or bad between environments. In this sense, the absence of control limits our interpretation, which leads us to conclude that ancient and modern survival scenarios produce similar recall performance.

Furthermore, we analyzed the effect of survival processing advantage on the use of medicinal plants to treat diseases in people who do not have continuous contact with this type of challenge or a dependence on this natural resource. Recruiting participants who do not experience the challenges present in simulated survival scenarios in the real world is common in experimental psychology studies (see studies [3, 8, 9, 63]). However, we argue this based on evidence that familiarity and previous experience with a recurring challenge in the environment influences how people perceive this risk [65, 66]. It was also observed in an experiment that medicinal plants previously known by the participants were prioritized in memory and were more easily remembered [22].

Thus, we suggest that future studies use adjusted protocols to test adaptive memory in real systems, in which the population has already faced a challenging event at some point or that deals with the challenge presented by the researcher on a relatively daily basis. In addition, we suggest that future studies examine if prior knowledge about the use of medicinal plants to treat disease, or of another natural resource, is a critical factor in the performance of the survival processing advantage effect on memory.

## Supporting information

S1 Appendix Average word evaluations and the proportion of recall. (DOCX)

S2 Appendix Processing scenarios used in experiment. (DOCX)

S3 Appendix Dataset. (XLSX)
