# Supplementary material for: Memory for medicinal plants remains in ancient and modern environments suggesting an evolved adaptedness
Source: PLoS One. 2021 Oct 25;16(10):e0258986. doi: 10.1371/journal.pone.0258986 (PMC8544875; doi:10.1371/journal.pone.0258986)
Supplement: S1 Appendix — (DOCX) [file pone.0258986.s001.docx]

**S1 Appendix. Average word evaluations and the proportion of recall (95% confidence interval) in coniferous forest, deciduous forest, desert, savanna, rainforest, tundra, and urban environments**.

|  |  | **Coniferous** | | **Deciduous** | | **Desert** | | **Savanna** | |
| --- | --- | --- | --- | --- | --- | --- | --- | --- | --- |
| Words | Translated words (english) | Rating | Recall | Rating | Recall | Rating | Recall | Rating | Recall |
| água | water | 5.00 | 0.26 | 5.00 | 0.43 | 4.70 | 0.83 | 3.96 | 0.86 |
| agulha | needle | 3.16 | 0.60 | 3.23 | 0.63 | 3.56 | 0.73 | 3.40 | 0.40 |
| algodão | cotton | 2.96 | 0.56 | 2.96 | 0.60 | 2.93 | 0.60 | 2.70 | 0.60 |
| animal | animal | 3.00 | 0.70 | 4.10 | 0.56 | 3.86 | 0.73 | 3.70 | 0.60 |
| aranha | spider | 2.50 | 0.30 | 1.56 | 0.26 | 1.80 | 0.30 | 1.66 | 0.20 |
| bíblia | bible | 1.66 | 0.43 | 1.90 | 0.40 | 1.80 | 0.40 | 1.93 | 0.60 |
| cama | bed | 2.56 | 0.36 | 2.66 | 0.40 | 2.90 | 0.53 | 2.60 | 0.63 |
| camisa | shirt | 4.16 | 0.40 | 4.23 | 0.43 | 3.73 | 0.53 | 3.46 | 0.46 |
| carro | car | 2.03 | 0.63 | 2.66 | 0.73 | 3.03 | 0.93 | 2.70 | 0.80 |
| charuto | cigar | 1.00 | 0.60 | 1.06 | 0.40 | 1.06 | 0.36 | 1.50 | 0.16 |
| dinheiro | money | 2.73 | 0.53 | 2.76 | 0.53 | 1.70 | 0.30 | 2.40 | 0.30 |
| fogo | fire | 4.06 | 0.30 | 4.26 | 0.30 | 4.16 | 0.40 | 3.70 | 0.63 |
| gás | gas | 3.03 | 0.33 | 3.33 | 0.26 | 2.96 | 0.23 | 2.13 | 0.53 |
| geladeira | fridge | 2.50 | 0.63 | 2.30 | 0.53 | 1.73 | 0.53 | 2.36 | 0.43 |
| jaula | cage | 1.26 | 0.16 | 1.50 | 0.16 | 1.80 | 0.16 | 2.00 | 0.23 |
| lápis | pencil | 1.96 | 0.36 | 2.03 | 0.30 | 1.80 | 0.26 | 1.73 | 0.36 |
| maquina | machine | 2.00 | 0.20 | 2.03 | 0.33 | 2.16 | 0.30 | 3.00 | 0.26 |
| natureza | nature | 4.80 | 0.70 | 4.73 | 0.63 | 4.70 | 0.60 | 4.43 | 0.63 |
| paisagem | landscape | 3.00 | 0.50 | 3.60 | 0.53 | 3.66 | 0.73 | 3.86 | 0.66 |
| papel | paper | 2.63 | 0.50 | 2.66 | 0.50 | 2.70 | 0.36 | 3.00 | 0.43 |
| pessoas | people | 4.16 | 0.60 | 4.30 | 0.33 | 4.30 | 0.73 | 4.56 | 0.53 |
| prato | plate | 1.53 | 0.40 | 1.46 | 0.56 | 1.73 | 0.33 | 1.40 | 0.80 |
| refrigerante | soda | 1.23 | 0.73 | 1.53 | 0.70 | 1.76 | 0.36 | 2.36 | 0.40 |
| remédio | remedy | 4.90 | 0.36 | 4.73 | 0.56 | 4.50 | 0.76 | 4.40 | 0.63 |
| saco | bag | 2.90 | 0.43 | 2.93 | 0.36 | 2.63 | 0.23 | 2.23 | 0.16 |
| sangue | blood | 3.80 | 0.26 | 4.20 | 0.30 | 4.06 | 0.53 | 4.10 | 0.73 |
| tampa | lid | 2.06 | 0.30 | 1.83 | 0.30 | 1.73 | 0.23 | 1.80 | 0.40 |
| terra | soil | 2.33 | 0.50 | 2.80 | 0.53 | 2.80 | 0.46 | 2.73 | 0.43 |
| tumulo | tomb | 1.26 | 0.16 | 1.10 | 0.10 | 1.36 | 0.10 | 1.63 | 0.10 |
| violão | guitar | 1.33 | 0.23 | 1.50 | 0.23 | 1.43 | 0.13 | 1.63 | 0.06 |
| vitamina | vitamin | 4.86 | 0.10 | 4.86 | 0.03 | 4.40 | 0.13 | 3.76 | 0.26 |
| xícara | tea-cup | 2.06 | 0.33 | 1.90 | 0.26 | 2.06 | 0.26 | 2.36 | 0.23 |
| **Averages** |  | **2.76** | **0.42** | **2.86** | **0.41** | **2.79** | **0.43** | **2.78** | **0.45** |

**Materials S1 (continuation)**

|  | | **Rainforest** | | **Tundra** | | **Urban** | |
| --- | --- | --- | --- | --- | --- | --- | --- |
| Words | Translated words (english) | Rating | Recall | Rating | Recall | Rating | Recall |
| água | water | 5.00 | 0.86 | 4.76 | 0.86 | 4.96 | 0.80 |
| agulha | needle | 3.10 | 0.70 | 3.43 | 0.76 | 3.40 | 0.70 |
| algodão | cotton | 3.13 | 0.56 | 3.36 | 0.66 | 3.56 | 0.56 |
| animal | animal | 3.93 | 0.76 | 3.80 | 0.73 | 3.80 | 0.73 |
| aranha | spider | 2.10 | 0.23 | 1.86 | 0.23 | 1.53 | 0.23 |
| bíblia | bible | 2.26 | 0.53 | 2.10 | 0.43 | 2.40 | 0.36 |
| cama | bed | 3.16 | 0.63 | 3.16 | 0.60 | 3.53 | 0.56 |
| camisa | shirt | 3.80 | 0.46 | 3.80 | 0.70 | 3.66 | 0.53 |
| carro | car | 1.93 | 0.80 | 2.73 | 0.90 | 3.23 | 0.90 |
| charuto | cigar | 1.06 | 0.20 | 1.20 | 0.30 | 1.20 | 0.23 |
| dinheiro | money | 1.50 | 0.50 | 2.43 | 0.36 | 4.23 | 0.36 |
| fogo | fire | 4.36 | 0.53 | 4.30 | 0.66 | 3.26 | 0.53 |
| gás | gas | 2.36 | 0.33 | 2.53 | 0.26 | 3.20 | 0.33 |
| geladeira | fridge | 1.43 | 0.60 | 2.20 | 0.60 | 2.83 | 0.50 |
| jaula | cage | 1.70 | 0.10 | 1.76 | 0.20 | 1.16 | 0.03 |
| lápis | pencil | 1.83 | 0.53 | 1.63 | 0.30 | 2.36 | 0.36 |
| maquina | machine | 1.83 | 0.40 | 2.16 | 0.26 | 2.06 | 0.26 |
| natureza | nature | 4.73 | 0.66 | 4.66 | 0.76 | 4.70 | 0.60 |
| paisagem | landscape | 3.73 | 0.63 | 3.46 | 0.80 | 3.36 | 0.56 |
| papel | paper | 2.43 | 0.36 | 2.70 | 0.33 | 2.73 | 0.43 |
| pessoas | people | 4.03 | 0.80 | 4.43 | 0.76 | 4.56 | 0.83 |
| prato | plate | 1.73 | 0.23 | 1.86 | 0.23 | 2.30 | 0.33 |
| refrigerante | soda | 1.30 | 0.26 | 1.46 | 0.33 | 1.33 | 0.50 |
| remédio | remedy | 4.73 | 0.80 | 4.63 | 0.83 | 4.83 | 0.73 |
| saco | bag | 3.00 | 0.26 | 2.66 | 0.33 | 3.06 | 0.13 |
| sangue | blood | 4.36 | 0.66 | 3.80 | 0.36 | 3.56 | 0.76 |
| tampa | lid | 1.76 | 0.13 | 1.56 | 0.26 | 2.00 | 0.36 |
| terra | soil | 3.06 | 0.36 | 2.90 | 0.50 | 3.33 | 0.46 |
| tumulo | tomb | 1.43 | 0.06 | 1.46 | 0.13 | 1.30 | 0.13 |
| violão | guitar | 1.30 | 0.20 | 1.56 | 0.20 | 1.50 | 0.26 |
| vitamina | vitamin | 4.53 | 0.16 | 3.96 | 0.20 | 4.63 | 0.13 |
| xícara | tea-cup | 1.80 | 0.90 | 2.00 | 0.23 | 2.30 | 0.26 |
| **Averages** | | **2.76** | **0.47** | **2.82** | **0.47** | **2.99** | **0.45** |
