# Supplementary material for: Memory for medicinal plants remains in ancient and modern environments suggesting an evolved adaptedness
Source: PLoS One. 2021 Oct 25;16(10):e0258986. doi: 10.1371/journal.pone.0258986 (PMC8544875; doi:10.1371/journal.pone.0258986)
Supplement: S2 Appendix — (DOCX) [file pone.0258986.s002.docx]

**S2 Appendix. Description of the simulated survival scenarios, texts of the situations, and the respective images of the environments.**

| **Environment** | **Text** | **Image^1^** |
| --- | --- | --- |
| Coniferous forest | *“Imagine that you are alone and sick in a coniferous forest, without basic materials for survival. In the coming days, you will need to find and use medicinal plants to treat this disease. We will show you a list of words, and we would like you to assess the relevance of each of these words in your attempt to treat the disease and survive in this environment”.* | 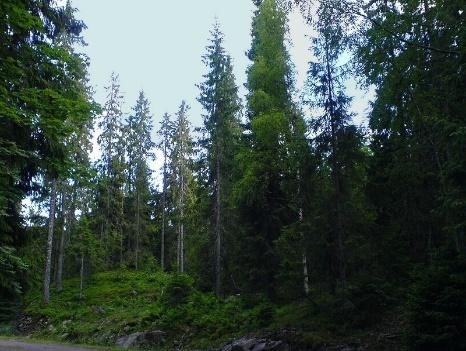 |
| Deciduous forest | *“Imagine that you are alone and sick in a deciduous forest, without basic materials for survival. In the coming days, you will need to find and use medicinal plants to treat this disease. We will show you a list of words, and we would like you to assess the relevance of each of these words in your attempt to treat the disease and survive in this environment”.* | 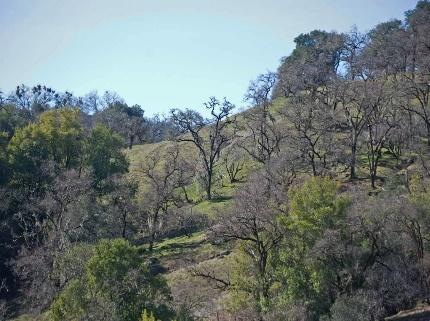 |
| Desert | *“Imagine that you are alone and sick in a desert, without basic materials for survival. In the coming days, you will need to find and use medicinal plants to treat this disease. We will show you a list of words, and we would like you to assess the relevance of each of these words in your attempt to treat the disease and survive in this environment”.* | 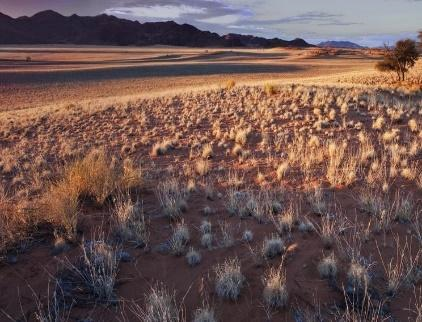 |
| Savanna | *“Imagine that you are alone and sick in a savanna, without basic materials for survival. In the coming days, you will need to find and use medicinal plants to treat this disease. We will show you a list of words, and we would like you to assess the relevance of each of these words in your attempt to treat the disease and survive in this environment”.* | 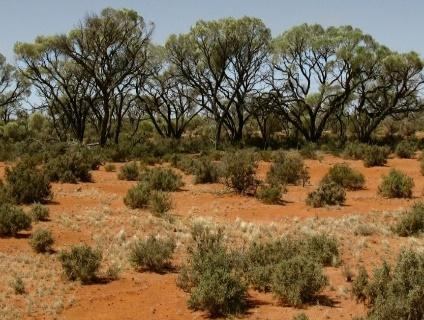 |
| Rainforest | *“Imagine that you are alone and sick in a rainforest, without basic materials for survival. In the coming days, you will need to find and use medicinal plants to treat this disease. We will show you a list of words, and we would like you to assess the relevance of each of these words in your attempt to treat the disease and survive in this environment”.* | 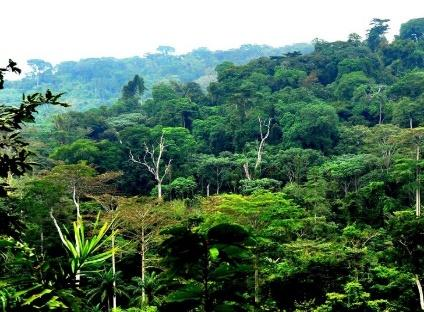 |
| Tundra | *“Imagine that you are alone and sick in a tundra, without basic materials for survival. In the coming days, you will need to find and use medicinal plants to treat this disease. We will show you a list of words, and we would like you to assess the relevance of each of these words in your attempt to treat the disease and survive in this environment”.* | 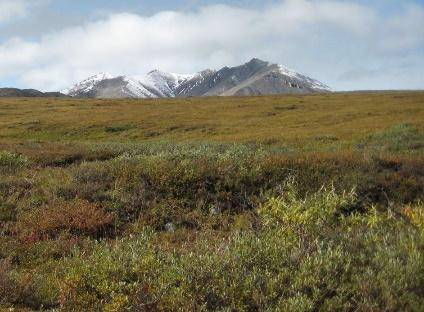 |
| Urban | *“Imagine that you are alone and sick in a city, without basic materials for survival. In the coming days, you will need to find and use medicinal plants to treat this disease. We will show you a list of words, and we would like you to assess the relevance of each of these words in your attempt to treat the disease and survive in this environment”.* | 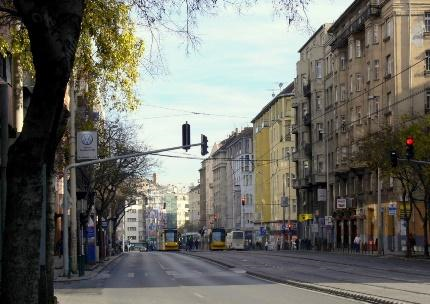 |

^1^ Source of free images:

**Savanna**

https://pixabay.com/en/photos/?q=savannah&image_type=&cat=&min_

height=&min_width=&order=popular&pagi=2%22

**Rainforest**

https://pixabay.com/en/photos/?q=tropical+forest&image_type=&cat=&min_height=&min_width=&order=popular&pagi=2

**Deciduous forest**

https://pixabay.com/en/photos/?q=deciduous+forest&image_type=&cat=

&min_height=&min_width=&order=popular&pagi=2

**Coniferous forest**

https://pixabay.com/en/photos/?q=coniferous+forest&image_type=&cat=

&min_height=&min_width=&order=popular&pagi=2

**Desert (deserto)**

https://pixabay.com/en/photos/?hp=&image_type=&cat=&min_width=&min_

height=&q=desert&order=popular

**Tundra**

https://pixabay.com/en/photos/?hp=&image_type=&cat=&min_width=&min_

height=&q=tundra&order=popular

**City**

https://pixabay.com/en/photos/?hp=

&image_type=&cat=&min_width=&min_height=&q=city&order=popular
